# Hybrid-2-Port-23/25-G-Pars-plana-Vitrektomie zur Entfernung von Öl höherer Viskosität

**DOI:** 10.1007/s00347-020-01114-5

**Published:** 2020-06-19

**Authors:** Lea Donata Priester, Panagiotis Laspas, Bernhard Stoffelns, Norbert Pfeiffer

**Affiliations:** Augenklinik und Poliklinik, Universitätsmedizin Mainz, Langenbeckstr. 1, 55131 Mainz, Deutschland

**Keywords:** Silikonölentfernung, Vitrektomie, Operationszeitverkürzung, Ölendotamponade, Netzhautchirurgie, Silicone oil removal, Vitrectomy, Shortening of operating time, Oil endotamponade, Retinal surgery

## Abstract

Wir stellen eine neue Hybridmethode einer kombinierten 23/25 G-Pars-plana-Vitrektomie (PPV) zur Ölentfernung (5700 Centistokes) vor und vergleichen sie mit einer 25 G PPV. Bei der Hybridmethode erfolgt die Infusion über einen 25-G-Zugang und die Absaugung des Öls über einen 23-G-Zugang. Es wurden die Operationszeit und der postoperative intraokuläre Druck (IOD) ausgewertet. Die Operationszeit konnte mit der Hybrid-Methode signifikant um 32,2 % reduziert werden. Postoperativ bestand kein IOD-Unterschied. Schlussfolgerung: Die Entfernung von Silikonöl durch einen 25-G-Zugang ist generell möglich, wobei die neue Hybridmethode die Operationszeit deutlich reduziert.

## Abstract (en)

This article presents a new hybrid method of a combined 2‑port 23/25G pars plana vitrectomy for removal of silicone oil (5700 centistokes) compared to a 25G vitrectomy. In this hybrid technique the infusion is performed through a 25G cannula and the oil removal through a 23G cannula. The duration of the surgery and the intraocular pressure (IOP) in both groups were compared. The oil removal using the hybrid technique was performed significantly faster with a reduction of the time by 32.2% but there was no difference in the IOP. In conclusion, silicone oil removal through a 25G cannula is generally possible but oil removal with the new hybrid technique is much faster.

Die Verwendung von Silikonöl in der Netzhautchirurgie ist seit 1962 [1] eine gängige Methode, um beispielsweise Netzhautablösungen [2] oder schwere Bulbustraumata [3] mittels Endotamponade zu therapieren. Allerdings kann eine intravitreale Langzeitendotamponade mittels Silikonöl zu schwerwiegenden Komplikationen, wie z. B. Sekundärglaukom, Katarakt oder Keratopathien [4], führen. Aus diesem Grund sollte das Silikonöl entfernt werden, sobald das Ziel der Tamponade erreicht und der Netzhautstatus stabil ist.

Zur Entfernung von Silikonöl kann man sich verschiedener Methoden bedienen, einer passiven und einer aktiven. Die passive Silikonölentfernung erfolgt als 2‑Port-Technik, bei der eine Infusion mit physiologischer Spüllösung über die Pars plana angeschlossen wird und das Silikonöl über eine Sklerotomieöffnung abfließt. Diese Methode benötigt mehr Zeit und erfordert eine Vergrößerung des Sklerotomiekanals auf mindestens 3 mm. Das wiederum führt häufiger zur okulären Hyoptensionen und diffusen Glaskörperblutungen.

Die heutzutage bevorzugte Variante ist die aktive Entfernung als 3‑Port-Technik über eine Pars-plana-Vitrektomie. Dort wird das Silikonöl aktiv aus dem Glaskörperraum abgesaugt. Initial wurde diese Methode mittels 20-G-Trokaren von O’Malley und Heintz etabliert [5].

Zwischenzeitlich wurden in den letzten Jahren die intraoperativen Techniken und intraokulären Zugänge weitestgehend mit dem Ziel modifiziert, die Glasköperoperationen atraumatischer zu gestalten und damit den postoperativen Heilungsprozess zu beschleunigen. Dazu gehört auch die Entwicklung und Vorstellung der Operationsmethode von nahtlosen transkonjunktivalen und transskleralen 23-G- und 25-G-Trokaren von Fujii und Eckardt in der Netzhautchirurgie [6–8].

Allerdings stellen die immer kleiner werdenden Zugänge von 23 G mit einem Durchmesser von 0,64 mm und 25 G mit einem Durchmesser von 0,5 mm ein physikalisches Problem bei der aktiven Entfernung von hochviskösem Silikonöl mit 5700 Centistokes dar. Betrachtet man dazu das Poisseuill-Gesetz, sieht man, dass die Flussrate mit immer kleiner werdendem Radius der Trokaröffnung um ein Vielfaches reduziert wird und darüber dann auch die Operationsdauer verlängert wird.

Mit dem Ziel, eine möglichst minimal-invasive und doch zeiteffiziente Methode zur Silikonölentfernung zu etablieren, entwickelte sich die Fragestellung der vorliegenden konsekutiven Fallstudie. Es wurde untersucht, ob eine Silikonölentfernung von 5700 Centistokes durch eine bisher noch nicht beschriebene Hybrid-2-Port-23/25-G-Methode eine veränderte Operationsdauer und einen veränderten postoperativen Augeninnendruck aufweist im Vergleich zur klassischen 25-G-Pars-plana-Vitrektomie. Es wurden bewusst diese Trokargrößen gewählt, da eine direkte, atraumatischere transkonjunktivale Applikation möglich ist ohne Eröffnung der Bindehaut und Kauterisierung der episkleralen Gefäße.

## Methoden

Es wurden 43 Patienten, die eine Pars-plana-Vitrektomie zur Entfernung einer Ölendotamponade höherer Viskosität (5700 Centistokes, Bausch & Lomb®, New York, NY, USA) erhielten, in diese konsekutive Fallserie eingeschlossen. Die Operationen wurden alle von einem erfahrenen Operateur durchgeführt.

Bei 21 Patienten fand die Ölentfernung mittels einer traditionellen 2‑Port-25-G-Pars-plana-Vitrektomie statt, bei den anderen 22 Patienten mittels einer bisher nicht beschriebenen Hybrid-2-port-23/25-G-Methode. Hierbei erfolgt die Infusion über einen 25-G-Zugang und die Absaugung der Ölendotamponade über einen 23-G-Trokar. Der 23-G-Trokar wurde zusätzlich und separat zum klassischen 25-G-Operationsset von Alcon® (Genf, Schweiz) verwendet. Der zusätzliche Trokar stammt von der Firma DORC® (Zuidland, Niederlande), wo er als Einzelprodukt erworben werden kann. Die Absaugung erfolgte mit einer Geräteeinstellung von 650 mm Hg, was einem intraokulären Druck von 25 mm Hg entspricht. Anschließend erhielten beide Gruppen nach klinikinternem Standard eine Naht aller beiden Sklerotomiezugänge. Als auszuwertende Parameter wurden sowohl die Zeitdauer der Operation als auch der intraokuläre Druck verglichen. Die Zeitdauer der Operation wurde anhand der Schnitt-Naht-Zeit im Operationsprotokoll abgelesen. Der postoperative Druck wurde mittels Goldmann-Tonometrie am ersten postoperativen Tag gemessen. Anschließend erfolgte die Auswertung mittels Statistikprogramm RStudio Open Source Edition Version 0.98.1062 (Boston, MA, USA) [9].

## Ergebnisse

**Figure 1:**
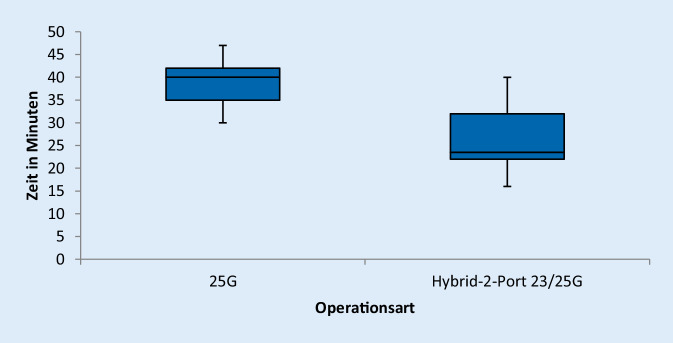

Es zeigte sich, dass die Operationsdauer mittels der Hybrid-2-Port-23/25-G-Pars-plana-Vitrektomie signifikant verkürzt werden konnte. So betrug die Operationszeit dort im Durchschnitt 26,18 min. Bei der klassischen 25-G-Pars-plana-Vitrektomie lag die durchschnittliche Operationszeit bei 38,62 min. Damit konnte die notwendige Operationsdauer um 32,2 % gesenkt werden, t (40) = 6,98; *p* < 0,01 (Abb. 1).

**Figure 2:**
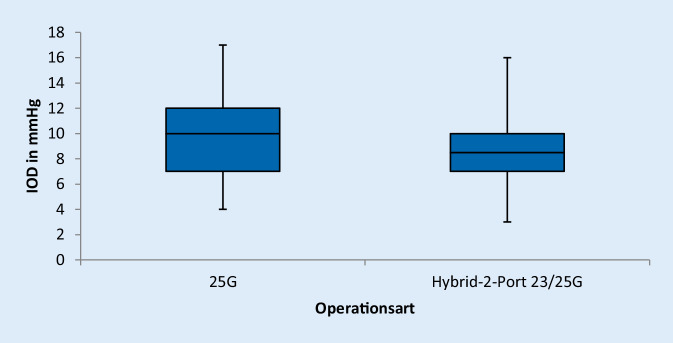

Der postoperative intraokuläre Druck unterschied sich bei beiden Gruppen kaum. Das Patientenkollektiv, das mittels der Hybridmethode operiert wurde, hatte einen durchschnittlichen postoperativen intraokulären Druck von 8,8 mm Hg, wohingegen die Vergleichsgruppe einen intraokulären Druck von 9,8 mm Hg aufwies; t (40) = 0,97; *p* = 0,34 (Abb. 2).

## Diskussion

In der vorliegenden Fallstudie wurde eine neue Methode zur Entfernung von hochviskösem Silikonöl mit 5700 Centistokes vorgestellt. Dabei handelt es sich um die Ölentfernung mittels eines Hybrid-2-Port-23/25-G-Zugangs, der mit der klassischen 2‑Port-25-G-Pars-plana-Vitrektomie verglichen wurde.

Grundsätzlich wurde zur Verminderung der Invasivität der Operation in beiden Fallgruppen die Silikonölentfernung lediglich mit einem 2‑Port-Zugang, statt wie bereits etabliert mit einem 3‑Port-Zugang, durchgeführt. Eine Anwendung von BIOM und Endo-Illumination ist bei der Ölentfernung selbst nicht unbedingt nötig. Erst zur finalen Beurteilung der Netzhaut, kann die Endo-Illumination über den vorhandenen zweiten Port verwendet werden.

Bei der Ölentfernung über 2 Zugänge kann das leichte Öl unter direkter Sicht durch das Mikroskop durch die Infusion nach oben geschoben werden. Mithilfe einer suffizienten Erweiterung der Pupille, passenden Rotation des Auges und Eindellung unterhalb des Aspirationstrokars ist es möglich, die Spitze des Trokars im Auge während der gesamten Operation durch das Mikroskop zu beobachten und die Ölentfernung direkt zu überprüfen, ohne Ölreste zu hinterlassen. Durch die Eigenschaft der höheren Viskosität und der damit einhergehenden erhöhten Oberflächenspannung des Silikonöls mit 5700 Centistokes verbleibt das Öl in einer homogenen Ölblase, ohne dass Ölbläschenreste entstehen. So sind bei diesem 2‑Port-Zugang lediglich die Infusion und Aspiration notwendig.

Außerdem konnte in dieser Fallstudie gezeigt werden, dass prinzipiell trotz geringen Durchmessers die Entfernung von hochviskösem Silikonöl mittels eines 25-G-Zugangs möglich ist. Die zu Beginn aufgestellte Hypothese, dass aufgrund des Poisseuill-Gesetzes die Operationsdauer bei einer 25-G-Pars-plana-Vitrektomie verlängert ist, konnte bestätigt werden. Damit ermöglicht die Hybrid-2-Port-Methode unter Verwendung eines zusätzlichen 23-G-Trokars, worüber das Öl aktiv abgesaugt wird, eine deutliche Verkürzung der Operationszeit und zugleich eine Reduktion die Invasivität der Operation durch einen 25-G-Zugang und einen 23-G-Zugang.

Bezüglich des postoperativen intraokulären Drucks gab es keinen signifikanten Unterschied zwischen beiden Gruppen. Das lässt sich v. a. dadurch erklären, dass bei beiden Gruppen der Verschluss der Sklerotomien mittels Naht erfolgte.

Zusammenfassend lässt sich sagen, dass die neu vorgestellte Operationsmethode, bei der ein 25-G-Zugang gewählt wurde und die aktive Ölentfernung über einen 23-G-Zugang erfolgte, eine weniger invasive und dennoch schnelle Methode darstellt, um suffizient hochvisköses Silikonöl aus dem Glaskörperraum zu entfernen.

## Fazit für die Praxis

Die vorgestellte 23/25-G-Hybridmethode stellt eine neue, weniger invasive Technik zur aktiven Ölentfernung dar, um dennoch schnell und suffizient Silikonöl aus dem Glaskörperraum zu entfernen.
